
JOURNAL INFORMATION
==============================
Journal ID: Wirtschaftsdienst

Wirtschaftsdienst

EISSN: 1613-978X

ARTICLE INFORMATION
==============================
PMCID: PMC9226276
DOI: 10.1007/s10273-022-3208-2
Article ID: 3208
Article version: 1
Subjects: Zeitgespräch

Die EZB muss die Inflation glaubwürdiger bekämpfen

The ECB Has To Tackle Inflation More Credibly

Horst Maximilian maximilian.horst@hhu.de

Stempel Daniel daniel.stempel@hhu.de

Neyer Ulrike ulrike.neyer@hhu.de

grid.411327.2 0000 0001 2176 9917 oeconomicum, Heinrich-Heine-Universität Düsseldorf, Universitätsstraße 1, 40225 Düsseldorf, Deutschland
Electronic publication date: 2022 Jun 24
Print publication date: 2022
Volume: 102
Issue: 6
First page: 426
Last page: 429
Copyright: © Der/die Autor:in 2022
License: Open Access: Dieser Artikel wird unter der Creative Commons Namensnennung 4.0 International Lizenz veröffentlicht (https://creativecommons.org/licenses/by/4.0/deed.de).
Open Access wird durch die ZBW — Leibniz-Informationszentrum Wirtschaft gefördert.
License URL: https://creativecommons.org/licenses/by/4.0/

Keywords: E31, E52, E58
issue-copyright-statement: © The Author(s) 2022

==============================

Die Inflation im Eurowährungsgebiet hat insbesondere aufgrund stark gestiegener Energiepreise historische Höchstwerte erreicht. Da das Eurowährungsgebiet ein Nettoimporteur von Energie ist, spricht man auch von importierter Inflation (Schnabel, 2022a). Es droht die Gefahr, dass sich diese hohen Inflationsraten in entsprechenden Inflationserwartungen niederschlagen und damit nicht nur zu einer Verfestigung der hohen Inflationsraten führen, sondern in einer gefährlichen Aufwärtsspirale der Preisentwicklung münden. Die EZB sollte deshalb jetzt klar und glaubwürdig kommunizieren, dass sie dieser Gefahr aktiv entgegenwirkt, und entsprechend handeln, denn die Kosten der Rückführung hoher Inflationsraten sind umso höher, je länger man wartet.

## Stark gestiegene Inflationsraten im Euroraum

Seit einem Jahr steigt die Inflationsrate im Euroraum stark an. Lag die Teuerungsrate gegenüber dem Vorjahresmonat gemäß des Harmonisierten Verbraucherpreisindexes (HVPI) im April 2021 mit 1,6 % noch unter der Zielinflationsrate der Europäischen Zentralbank (EZB),[1] erklomm sie im April dieses Jahres mit 7,4 % einen Rekordwert (vgl. Abbildung 1). Eine ähnliche Entwicklung lässt sich auch in Deutschland beobachten.

1 Bis Juli 2021 spezifizierte die EZB ihr Ziel der Preisstabilität als eine Inflationsrate von mittelfristig unter, aber nahe 2 %. Nach der Überarbeitung der geldpolitischen Strategie wurde das Inflationsziel auf symmetrische 2 % festgelegt.

## Ursachen für die aktuelle Inflationsentwicklung

Der Anstieg der Inflationsrate in der zweiten Jahreshälfte 2021 hat angebots- und nachfrageseitige Ursachen. Expansive fiskal- und geldpolitische Maßnahmen sowie der Impffortschritt gegen COVID-19 und die damit verbundene Rückführung vieler Maßnahmen zur Eindämmung der Pandemie führten zu einem Anstieg in der aggregierten Nachfrage. Dadurch kam es zu einer schnellen Erholung der europäischen Wirtschaft und zu einem Anstieg der Inflation. Dieser Anstieg fiel auch deshalb so hoch aus, weil die Preise im Vorjahreszeitraum pandemiebedingt besonders niedrig waren. Dieser sogenannte Basiseffekt wurde von angebotsseitigen Lieferengpässen, insbesondere von Rohstoffen, weiter verstärkt. Diese Angebotsschocks lassen sich ebenfalls auf die Coronapandemie zurückführen: Einerseits führte der weltweite Anstieg in der Nachfrage aufgrund der Rückführung von Pandemiemaßnahmen zu weltweiten Überlastungen der Logistik, insbesondere von Häfen. Andererseits kam es aufgrund der restriktiven „Zero-Covid"-Politik in China zu erheblichen Einschränkungen der Logistik in chinesischen Häfen.

Diese pandemiegetriebenen Effekte veranlassten viele Ökonom:innen dazu, den Anstieg in der Inflationsrate als vorübergehend anzusehen. Entsprechend lagen die EZB-Prognosen für die Inflationsrate im Jahr 2022 im September 2021 noch bei 1,7 %, welche im Dezember 2021 auf 3,2 % erhöht wurde (EZB, 2022a).

Allerdings verzeichnete die Inflationsrate im Euroraum im Frühjahr dieses Jahres weitere starke Anstiege. Dies ist insbesondere auf den Krieg in der Ukraine zurückzuführen, welcher die Auswirkungen der beschriebenen Angebotsschocks verstärkt. Insbesondere der Anstieg der Energiepreise impliziert eine Erhöhung des HVPI, sowohl durch eine direkte Belastung von Haushalten aufgrund der Verteuerung von Elektrizität oder Gas (um 42,8 % in der Eurozone) als auch durch eine indirekte Belastung aufgrund erhöhter Kosten für Unternehmen, die diese über Preissteigerungen an ihre Kundschaft weitergeben.

Fasst man die Definition der importierten Inflation weiter, also als Inflation, die durch Handel auftritt, können die bereits erfolgten kontraktiven geldpolitischen Maßnahmen der US-amerikanischen Notenbank (Leitzinserhöhungen, Verringerung des Wertpapierbestands in der Zentralbankbilanz) zusätzliche importierte Preissteigerungen induzieren. Die Zinserhöhungen führen zu einer Abwer-

Abbildung 1

**Inflationsrate im Euroraum und in Deutschland**

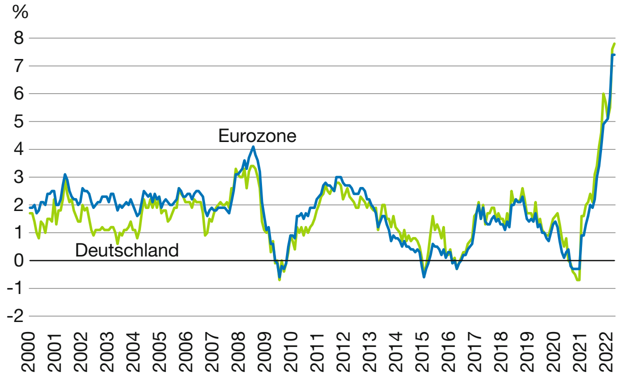

Quelle: Eurostat.

tung des Euros im Vergleich zum Dollar (im Juni 2021 noch 1,2 US-$, aktuell 1,07 US-$ je Euro) und somit zu erhöhter Nachfrage nach Produkten in der Eurozone.

## Verfestigung erhöhter Inflationsraten

Die Inflationsentwicklung ist für die EZB besonders besorgniserregend, wenn sie nicht kurzfristig und vorübergehend auftritt, sondern sich in Preissetzung und Erwartungen verfestigt. Zwar prognostiziert die EZB einen Rückgang der Inflationsrate auf 2,1 % schon im Jahr 2023 (EZB, 2022b), allerdings weisen typische Indikatoren auf eine Verfestigung der erhöhten Inflationsraten hin. Solche Indikatoren sind insbesondere Umfragen zu den Erwartungen hinsichtlich der zukünftigen Inflationsentwicklung, die Entwicklung der Erzeugerpreise und das Niveau der Tarifabschlüsse. Zwar stimmen die Erwartungen professioneller Prognostiker:innen mit den Prognosen der EZB in etwa überein (EZB, 2022c), allerdings zeigen andere Erhebungen innerhalb der Eurozone, dass sich die Inflationserwartungen nicht überall auf diesem niedrigen Niveau bewegen. In Deutschland erwarten Ökonom:innen beispielsweise eine deutlich erhöhte Inflationsrate mindestens bis ins Jahr 2026 (Ökonomenpanel, 2022). Haushaltsbefragungen der Bundesbank (2022) liefern ähnliche Ergebnisse.

Weiteren Grund zur Sorge bereiten die Entwicklungen der Erzeugerpreise im Euroraum. Im März 2022 lagen die Erzeugerpreise in der Industrie im Euroraum 28,3 % über dem Wert des Vorjahresmonats.

Zuletzt spiegeln die Tarifabschlüsse in der Eurozone eine mögliche Verfestigung der Inflationsraten wider. In Deutschland lassen sich beispielsweise einige Tarifabschlüsse mit Lohnanstiegen über einem Niveau von 3 % beobachten (Energiewirtschaft, privates Transport- und Verkehrsgewerbe, Versicherungsgewerbe). Tarifabschlüsse mit Lohnanstiegen von bis zu 3 % sind, unter Annahme eines Produktivitätswachstums von 1 %, mit dem Inflationsziel vereinbar (Lane, 2022). Mögliche weiter steigende Löhne in zukünftigen Tarifabschlüssen würden die Gefahr einer Lohn-Preis-Spirale erhöhen.

Die EZB-Präsidentin Christine Lagarde wies in der Pressemitteilung der EZB zu den geldpolitischen Beschlüssen im April 2022 auf die deutlich gestiegene Inflationsrate im Euroraum hin. Des Weiteren machte sie auf den sektorübergreifenden, intensivierten Inflationsdruck sowie auf potenziell schnelle Lohnanstiege aufmerksam (Lagarde, 2022a). Schwer verständlich ist daher die zögerliche Kommunikation der EZB hinsichtlich einer möglichen Straffung der expansiven Geldpolitik.

## Aktuelle Geldpolitik der EZB

Die extrem expansiv ausgerichtete Geldpolitik der EZB ist zum einen durch die sehr niedrigen Leitzinsen (vgl. Abbildung 2) und zum anderen durch die immer noch positiven Netto-Wertpapierkäufe (Quantitative Easing) als ursprüngliche Reaktion auf eine lang anhaltende Phase (zu) niedriger, teils negativer Inflationsraten gekennzeichnet. Dabei ist die Einführung der Wertpapierkäufe im März 2015 insbesondere auf das Erreichen der Zinsuntergrenze bei den Leitzinsen zurückzuführen. Das Ziel der Wertpapierkäufe besteht darin, die langfristigen Zinsen zu senken und final die Inflation zu erhöhen. Im Zuge der Coronapandemie wurden die Wertpapierkäufe im März 2020 in Form des Pandemic Emergency Purchase Programme (PEPP) zusätzlich massiv ausgeweitet.

Als Indikator für den Expansionsgrad der Geldpolitik kann der sogenannte Schattenzins herangezogen werden. Ökonomisch stellt der Schattenzins denjenigen theoretischen Zins dar, der sich ergäbe, wenn die Zinsuntergrenze nicht existieren würde und statt Wertpapierkäufen weiterhin konventionelle Leitzinssenkungen durchgeführt würden.[2] Wie Abbildung 2 zeigt, ist der Expansionsgrad der Geldpolitik aufgrund der Wertpapierkäufe in den vergangenen Jahren stetig gestiegen und entspräche aktuell immer noch einem Leitzinsniveau von ca. minus 5 %. In Anbetracht der Kombination aus den derzeitig hohen Inflationsraten und den potenziellen Inflationsverfestigungen erscheint dieser Expansionsgrad nicht mehr angemessen.

2 Wu und Xia (2016, 2020) haben das Konzept des Schattenzinses entwickelt, um die beiden expansiven geldpolitischen Instrumente, Leitzinssenkungen und Wertpapierankäufe, quantitativ vergleichbar zu machen.

Abbildung 2
**EZB-Leitzinsen und Schattenzins (Wu und Xia)**

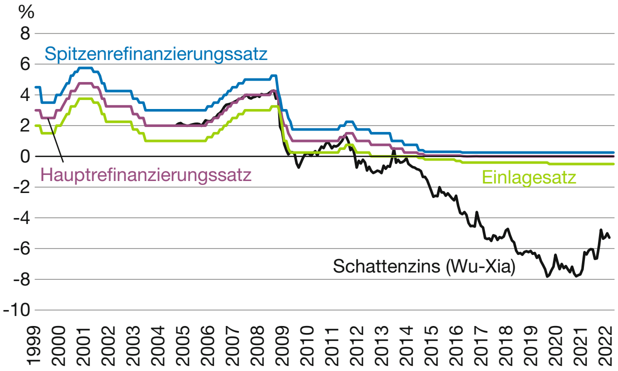

Quelle: Eurosystem.

Somit stehen aktuell zwei geldpolitische Instrumente zur Inflationsbekämpfung im Fokus. Die EZB hat die Möglichkeit, zunächst (i) ihre (Netto-)Wertpapierkäufe einzustellen, (ii) mit Leitzinserhöhungen zu beginnen oder (iii) eine Kombination aus beidem durchzuführen. Die EZB hat mehrfach betont, das sogenannte Sequencing, also den Ausstieg aus der ultralockeren Geldpolitik so zu gestalten, dass es keine Zinserhöhungen geben wird, bevor die Netto-Wertpapierkäufe nicht eingestellt worden sind (Schnabel, 2021, 2022b). Vor diesem Hintergrund wurde Ende März dieses Jahres begonnen, die Wertpapierkäufe zu reduzieren. Dies dürfte ein Grund für den leichten Anstieg des Schattenzinses am aktuellen Rand sein (vgl. Abbildung 2). Im dritten Quartal dieses Jahres sollen die Netto-Wertpapierkäufe vollständig eingestellt werden (EZB, 2022d). Aus den bis dato letzten geldpolitischen Beschlüssen der EZB-Ratssitzung vom 14. April 2022 ging noch hervor, dass Leitzinsänderungen erst für „einige Zeit nach" dem Zeitpunkt des Einstellens der Netto-Wertpapierkäufe angekündigt würden (EZB, 2022d). Nach neuesten Ankündigungen Christine Lagardes im Mai 2022 werden die Netto-Wertpapierkäufe jedoch „sehr früh im dritten Quartal" enden und die EZB wahrscheinlich „einige Wochen später" im Juli mit ersten Zinserhöhungen beginnen. Darüber hinaus sei es wahrscheinlich, dass der Einlagesatz im September den negativen Bereich erstmalig seit mehr als acht Jahren wieder verlassen werde (Lagarde, 2022b, 2022c). Offiziell verkündet wurden diese Maßnahmen bislang jedoch nicht. Mit genaueren Informationen ist bei der nächsten EZB-Ratssitzung am 9. Juni 2022 zu rechnen.

## Herausforderungen für die EZB

Die Herausforderungen für die EZB sind demnach derzeit immens: Die hohe, in erster Linie importierte Inflation droht die Inflationserwartungen zu entankern. Dieses kann nicht nur zu einer Verfestigung der hohen Inflationsraten führen, sondern über Zweitrundeneffekte auch in einer gefährlichen Aufwärtsspirale der Preisentwicklung münden. Die angebotsseitigen Probleme (hohe Energiepreise, Lieferengpässe) bewirken jedoch nicht nur einen starken Aufwärtsdruck auf die Inflation, sondern gefährden auch die eingesetzte Erholung der Wirtschaft nach dem Einbruch in der Coronapandemie. Die EZB sieht sich mit den typischen Folgen eines negativen Angebotsschocks konfrontiert: steigende Inflation bei gleichzeitigem Rückgang der gesamtwirtschaftlichen Produktion. Zudem ist ein Teil der aktuellen Preissteigerungen importiert und entzieht sich damit der direkten geldpolitischen Steuerung (insbesondere die direkten Belastungen für Haushalte durch den Anstieg der Energiekosten).

Stellt die EZB die Netto-Wertpapierkäufe ein und erhöht die Leitzinsen, verschlechtern sich die Finanzierungsbedingungen im Euroraum. Kredite werden teurer. Dieses reduziert den Druck auf die Preise, gefährdet jedoch zusätzlich die wirtschaftliche Erholung im Euroraum. Hinzu kommt, dass die mit der Straffung der Geldpolitik verbundenen höheren Zinsen die Staatshaushalte insbesondere der ohnehin hochverschuldeten Euroländer wie Griechenland, Italien, Portugal und Spanien weiter belasten. Auch können Zinserhöhungen zu Problemen im Bankensektor führen. Banken betreiben Fristentransformation, das heißt, sie verschulden sich kurzfristig und legen Mittel langfristig an. Die durch die Geldpolitik ausgelösten Zinserhöhungen bewirken dann, dass die Zinskosten der Banken stärker steigen als ihre Zinserträge. Ferner können die mit dem Zinsanstieg verbundenen Kursverluste von Wertpapieren Bankbilanzen belasten.[3]

Trotz dieser Gefahren muss die EZB die Inflation bekämpfen. Dazu bedarf es einer klaren und glaubwürdigen Kommunikation und einer entsprechenden Straffung der Geldpolitik. Die EZB muss glaubwürdig vermitteln, dass sie die Inflation bekämpft, um einer Entankerung der Inflationserwartungen entgegenzuwirken. Je länger sie wartet, desto teurer wird die letztendlich notwendige Rückführung der hohen Inflationsraten. Ein Beispiel für eine mit immens hohen Kosten verbundene Disinflation ist die Inflationsbekämpfung in den USA Anfang der 1980er Jahre von Paul Volcker, dem damaligen Präsidenten der US-Zentralbank. Seine stark kontraktiv ausgerichtete Geldpolitik führte zu einem Rückgang der Inflationsrate von 13,5 % auf 3,2 %

3 Fabio Panetta, Mitglied des EZB-Direktoriums, beschreibt ausführlich die Risiken, die mit einer „Rücknahme der Akkomodierung" also einer weniger stark ausgeprägten expansiven Geldpolitik verbunden sind (Panetta, 2022).

innerhalb von drei Jahren. Sie bewirkte jedoch auch, dass die Wirtschaft in eine tiefe Rezession rutschte.

Die EZB sollte also aktiv die Zinswende einleiten, das heißt das Zinsniveau im Euroraum erhöhen, indem sie die Netto-Wertpapierkäufe mindestens beendet und die Leitzinsen erhöht. Die Erhöhung des Zinsniveaus impliziert eine Verringerung der gesamtwirtschaftlichen Nachfrage und reduziert damit den Druck auf die Preise. Ferner wertet der Euro gegenüber anderen Währungen auf, insbesondere gegenüber dem US-Dollar. Dies hat einen dämpfenden Effekt auf die importierte Inflation.

Die Einstellung der Netto-Wertpapierkäufe und eine Zinserhöhung hat die EZB für das dritte Quartal dieses Jahres bereits angekündigt. Unklar ist bislang, wie die Zinserhöhungen ausgestaltet werden. Erwartet wird für Juli eine Erhöhung des Einlagesatzes um 0,25 Prozentpunkte auf minus 0,25 % und ein Verlassen des negativen Zinsbereichs im September. Warum verlässt die EZB den negativen Zinsbereich nicht bereits im Juli? Sie würde damit glaubwürdiger vermitteln, dass sie gewillt ist, ihr Mandat zu erfüllen, ohne dass sie massiv auf die geldpolitische Bremse drückt. Die Geldpolitik ist mit einem Leitzins von null immer noch extrem expansiv ausgerichtet.

Die EZB hat ein eindeutiges geldpolitisches Mandat, nämlich für Preisniveaustabilität zu sorgen. Dies impliziert auch eine mit Kosten verbundene Inflationsbekämpfung. Diese sollte zwar mit Augenmaß sein, das heißt keine massiven, abrupten Zinserhöhungen, aber die EZB sollte *erstens* aktiv ihr geldpolitisches Instrumentarium zur Inflationsbekämpfung einsetzen und *zweitens* die Bereitschaft dazu klarer und glaubwürdiger kommunizieren als bisher.

**Title:** ***The ECB Has To Tackle Inflation More Credibly***

Inflation rates in the euro area have reached historic highs due in large part to high energy prices. As the euro area is a net importer of energy, one refers to this inflation as imported inflation. There is a danger that these high inflation rates will become entrenched in inflation expectations. This would not only imply that high inflation rates will persist but it could also cause a dangerous upward price spiral. Consequently, the ECB should communicate more clearly and more credibly that it will counteract this danger and act accordingly as the costs of disinflation will remain higher the longer the ECB waits to act.

Dr. Maximilian Horst und Daniel Stempel, Ph. D. sind wissenschaftliche Mitarbeiter an der Wirtschaftswissenschaftlichen Fakultät der Heinrich-Heine-Universität Düsseldorf.

Prof. Dr. Ulrike Neyer ist Professorin für Volkswirtschaftslehre, insbesondere Monetäre Ökonomik an der Heinrich-Heine-Universität Düsseldorf.


Literatur

Bundesbank (2022), Inflationserwartungen: Studie zu Erwartungen von Privatpersonen in Deutschland, https://www.bundesbank.de/de/bundesbank/forschung/erwartungsstudie/inflationserwartungen-849084 (27. Mai 2022).
EZB (2022a), Past Macroeconomic Projections, https://www.ecb.europa.eu/mopo/strategy/ecana/html/table.de.html (27. Mai 2022).
EZB (2022b), ECB Staff Macroeconomic Projections for the Euro Area, https://www.ecb.europa.eu/pub/projections/html/index.en.html (27. Mai 2022).
EZB (2022c), ECB Survey of Professional Forecasters, Q2 2022, https://www.ecb.europa.eu/stats/ecb_surveys/survey_of_professional_forecasters/html/index.en.html (27. Mai 2022).
EZB (2022d), Geldpolitische Beschlüsse, Pressemitteilung, https://www.ecb.europa.eu/press/pr/date/2022/html/ecb.mp220414∼d1b76520c6.de.html (27. Mai 2022).
Lagarde, C. (2022a), Monetary Policy Statement, Pressekonferenz, https://www.ecb.europa.eu/press/pressconf/shared/pdf/ecb.ds220414∼2d6ffb3a83.en.pdf (27. Mai 2022).
Lagarde, C. (2022b), Challenges Along Europe’s Road, Rede bei der internationalen Konferenz anlässlich des 30. Geburtstags der Slowenischen Zentralbank, Ljubljana, https://www.ecb.europa.eu/press/key/date/2022/html/ecb.sp220511∼4c8d4500f6.en.html (27. Mai 2022).
Lagarde, C. (2022c), Monetary Policy Normalisation in the Euro Area, EZB Blog Beitrag, Frankfurt am Main, https://www.ecb.europa.eu/press/blog/date/2022/html/ecb.blog220523∼1f44a9e916.de.html (27. Mai 2022).
Lane, P. R. (2022). The Euro Area Outlook: Some Analytical Considerations, Rede, https://www.ecb.europa.eu/press/key/date/2022/html/ecb.sp220505∼dcbd30ecb6.en.html (27. Mai 2022).
Ökonomenpanel (2022), Ökonomenpanel von ifo und FAZ, https://www.ifo.de/node/68326 (27. Mai 2022).
Panetta, F. (2022). Normalisierung der Geldpolitik in unnormalen Zeiten, Rede im Rahmen der Policy Lecture-Reihe des SAFE Policy Center an der Goethe-Universität und des Centre for Economic Policy Research (CEPR), Frankfurt am Main, https://www.ecb.europa.eu/press/key/date/2022/html/ecb.sp220525∼eef274e856.de.html (27. Mai 2022).
Schnabel, I. (2021), Asset Purchases: From Crisis to Recovery, Rede bei der “Annual Conference of Latvijas Banka on Sustainable Economy in Times of Change”, Frankfurt am Main, https://www.ecb.europa.eu/press/key/date/2021/html/ecb.sp210920∼ae2c7412dc.en.html (27. Mai 2022).
Schnabel, I. (2022a), The Globalisation of Inflation, Rede bei einer Konferenz organisiert von der Österreichischen Vereinigung für Finanzanalyse und Asset Management, Wien, https://www.ecb.europa.eu/press/key/date/2022/html/ecb.sp220511_1∼e9ba02e127.en.html (27. Mai 2022).
Schnabel, I. (2022b), Unwinding QE — Finding the Right Sequence, Rede bei der “First Annual Bank of England Agenda for Research (BEAR) Conference”, Frankfurt am Main, https://www.ecb.europa.eu/press/key/date/2022/html/ecb.sp220224∼232cb567cd.en.html (27. Mai 2022).
Wu C J Xia F D N Measuring the Macroeconomic Impact of Monetary Policy at the Zero Lower Bound Journal of Money, Credit and Banking 2016 48 253 291 10.1111/jmcb.12300
Wu C J Xia F D N Negative Interest Rate Policy and Yield Curve Journal of Applied Econometrics 2020 35 6 653 672 10.1002/jae.2767
